# The United Nations Office on Drugs and Crime’s Efforts to Strengthen a Culture of Prevention in Low- and Middle-Income Countries

**DOI:** 10.1007/s11121-020-01088-5

**Published:** 2020-01-17

**Authors:** Hanna Heikkilä, Wadih Maalouf, Giovanna Campello

**Affiliations:** 1 Finnish Association for Substance Use Prevention, United Nations Office on Drugs and Crime, Vienna, Austria; 2 United Nations Office on Drugs and Crime, Vienna, Austria

**Keywords:** Evidence based programs, Evidence based policies, Dissemination, Scaling up, Culture of prevention, Global prevention practices, Global monitoring systems, Global drug policy

## Abstract

This article discusses how decision-makers can be supported to strengthen a culture of prevention. This article presents an example of the United Nations Office on Drugs and Crime’s (UNODC) work to engage with decision-makers to create readiness, demand, and capacity for evidence-based prevention programming among them, particularly in low- and middle-income countries. First, we utilized two of the UNODC’s data sources to describe the context where the UNODC’s prevention efforts take place. Analysis of the first dataset on prevention activities implemented globally revealed a gap in translating evidence into practice on a global scale. The second dataset consisted of UNODC policy documents mandating and guiding global action to address substance use. The analysis showed that at the level of political frameworks, prevention is gradually gaining more attention but is still frequently left in the shadow of health- and law enforcement-related issues. In addition, these guiding documents did not reflect fully the current scientific understanding of what constitutes an effective prevention response. Against this background, the feasibility of the UNODC’s efforts to bridge the science–practice gap in the field of prevention was discussed by presenting the results from the UNODC’s regional capacity-building seminars focused on the role of monitoring and evaluation in prevention programming. The results showed potential of this capacity building to affect the attitudes and knowledge of targeted decision-makers. Such efforts to increase decision-makers’ readiness and ultimately their endorsement, adoption, and ongoing support of evidence-based preventive interventions should be continued and intensified.

**Electronic supplementary material:**

The online version of this article (10.1007/s11121-020-01088-5) contains supplementary material, which is available to authorized users.

Increasing efforts to use scientific knowledge to inform policymaking in the fields of health, education, and welfare have been observed in various contexts (Crowley et al. 2018; Ritter et al. 2018). In substance use prevention, the need to disseminate effective practices is pressing. Substance use continues to have a significant global toll on health and social and economic development (Degenhardt et al. 2013), and its prevention is high on the international political agenda. A high-level mandate for prevention is provided in the Agenda 2030 for Sustainable Development, adopted by all United Nations Member States as a call for action for achieving peace and prosperity for the humankind and the planet. The Sustainable Development Goal 3.5 specifically calls for strengthening measures to prevent substance use. (United Nations Department of Economic and Social Affairs n.d.) This development agenda together with the advancements in translational research provide opportune momentum for supporting a culture of prevention on a global scale via facilitating the translation of scientific understanding into prevention practices.

The role of the United Nations Office on Drugs and Crime (UNODC), inter alia, is to support all its member states, especially low- and middle-income countries (LMICs), in fulfilling their commitment to eliminate or significantly reduce the non-medical use of controlled substances (Commission on Narcotic Drugs 2009). We examine the feasibility of the UNODC’s efforts to engage decision-makers under its so-called top-down approach to advance the large-scale, global utilization of evidence-based policies and programs. To contextualize the UNODC’s efforts and illustrate how much remains to be done globally to advance a culture of prevention, we point to the translation gap in global prevention programming and the global political frameworks guiding these prevention practices. To do so, we use two datasets derived from the UNODC’s context. Against this background, the UNODC’s efforts to engage with decision-makers to facilitate bridging this gap are discussed.

Although the UNODC uses a variety of modalities to engage with decision-makers, we focus on capacity building targeted at building national-level decision-makers’ capacities, especially in relation to evaluation in prevention programming. We discuss the feasibility of this capacity-building exercise to create readiness among national policymakers to facilitate evidence-based prevention. Our analyses of the feasibility of this capacity building exercise are based on theory of change (De Silva et al. 2014) that takes as a starting point the barriers identified in the translational research and assumes that policymakers’ knowledge, attitudes, and intentions reflect their readiness to support evidence-based prevention. Thus, the knowledge, attitudes, and intentions of the policymakers are used as proxy indicators for readiness to analyze the likelihood that this training will achieve its aims.

## Framework for Promoting a Culture of Prevention

In a recent article, Parra-Cardona et al. (2018) grounded their understanding of a culture of prevention on the definition of the aims of prevention science provided by the Society for Prevention Research’s standards of knowledge (Biglan et al. 2011): to improve public health by (1) identifying malleable risk and protective factors; (2) assessing the effectiveness of preventive interventions targeting these risk factors; and (3) identifying optimal means to disseminate these interventions. Accordingly, a strong culture of prevention builds on an empirically derived understanding of the determinants of substance use behavior. This culture also develops, disseminates, and sustains effective interventions for prevention of substance use. When talking about strengthening a culture of prevention, we essentially mean further translation of prevention science into real-life practices and policies.

By itself, evidence of effective practices is not sufficient to change existing practices. The current literature identifies various barriers and enablers that can impede or aid the adoption of evidence-based practices. These factors include the needs, beliefs, values, priorities, and skills of decision-makers and the social norms and other contextual aspects that lend acceptability to reasoning and decision-making in different contexts (Hassmiller Lich et al. 2016; Oliver et al. 2014; Pentz et al. 2004; Ward et al. 2012; Wathen and MacMillan 2018). We understand these factors that work to legitimize or de-legitimize decision-making concerning prevention practices and systems to constitute a key layer on which to build a strong culture of prevention.

Applied prevention science has mapped the stages of knowledge translation needed to develop, test, scale-up, and sustain impactful prevention strategies. Understanding this translation process helps guide the integration of evidence into practices, policies, and eventually complex, real-world prevention systems. In addition to describing the translation process, concrete strategies for facilitating this translation have been identified in translational research. As well as influencing the attitudes and understanding of policymakers and the norms and cultures within which they operate, such strategies in the literature point to the importance of tailoring and packaging research specifically for policymakers and creating two-way communication with them (Aldridge et al. 2016; Crowley et al. 2018; Hassmiller Lich et al. 2016; Oliver et al. 2014; Pentz et al. 2004; Wathen and MacMillan 2018).

Many opportunities to utilize evidence in prevention practices and policymaking have yet to be realized; gaps have been identified, especially toward the end stages of the translation process. Good documentation exists concerning the first stages of the translation process, including creating and testing interventions (stages 0–2) and adapting and transferring them into real-world settings without losing their efficacy or effectiveness (stage 3). However, the effective scale-up and institutionalization of evidence-based prevention (stage 4) is a less developed research field. This is especially true in regard to the last stage (stage 5) of the translation process, which is geared toward influencing global policies and generating environmental change across multiple cultures and societies. This last stage includes shaping international behavioral health priorities and agendas and supporting large-scale population-level shifts in well-being via evidence-based practices (Crowley et al. 2018; Fishbein et al. 2016; Hassmiller Lich et al. 2016; Spoth et al. 2013).

## Context of the UNODC Prevention Program

The Commission on Narcotic Drugs (CND) is the governing body of the UNODC and serves as the policymaking body that formally discusses and agrees upon the global drug policy agenda. The resolutions and decisions made by the member states’ representatives on the CND provide guidance for addressing the issue of drugs to all the member states and the UNODC. While adopted by consensus, these resolutions are not binding but merely guide the member states and provide a normative framework for their actions. In the present article, the work of the UNODC is portrayed against developments in global cooperation on drugs over 2009–2017. The heads of the UNODC member states set goals for this period for countering the world drug problem in the 2009 Political Declaration (CND 2009) calling for greater commitment on drug demand reduction as part of these shared goals. They also adopted a revised annual report questionnaire (ARQ; United Nations Office on Drugs and Crime n.d.) to track and report their own progress in meeting these commitments. The questionnaire is an instrument for the member states to voluntarily report their own drug policy-related practices and policies, and the UNODC collates this information. We first present data from the ARQ to show how substance use prevention practices evolved globally over the years 2009–2015. Next, data from the CND’s resolutions, which are political documents mandating and guiding global action on prevention, are used to illustrate how the political frameworks guiding global action reflect the current scientific understanding of what constitutes an effective prevention response.

The International Standards on Drug Use Prevention (UN 2015), initially launched in 2013, summarizes the available evidence on what constitutes an effective prevention response and describe the different types of evidence-supported prevention approaches (Campello et al. 2014). The standards are used as a lens through which to analyze the available data on globally implemented prevention practices to establish a picture of the alignment of these practices with current scientific understanding.

### Quality and Coverage of Prevention Practices Globally

#### Materials and Methods for Analyzing the Global State of Prevention Practices

The ARQ, which covers a wide spectrum of drug policy-related issues, consists of four parts on (1) legislative and institutional frameworks; (2) comprehensive approaches to drug demand reduction and supply; (3) extent and patterns of drug use; and (4) extent and patterns of trends in drug crop cultivation and drug manufacture and trafficking. The second part of the ARQ requests information on the prevention and early intervention responses implemented in the reporting year by each member state, among other demand, and supply reduction issues. We describe trends in the prevention practices implemented globally by presenting data from this part of the ARQ across its three reporting cycles: 2010–2011 (reported in 2012), 2012–2013 (reported in 2014), and 2014–2015 (reported in 2016).

The ARQ section on prevention offers a list of different prevention approaches from which the member states can choose to indicate which of these readily defined types of prevention activities they are implementing. The member states are also asked to provide information on the size of the population they are reaching with these activities^1^ and whether these activities have been evaluated. For the purpose of presenting an informative overview of the collected data in this article, we categorized these prevention types according to the level of evidence by which they were deemed to be supported per the International Standards on Drug Use Prevention (UN 2015). The categories of prevention activities were as follows: (1) responses with no or limited evidence of efficacy, including media campaigns, provision of alternative activities, and dissemination of information on the dangers of drugs; (2) responses with good levels of efficacy, such as workplace prevention programs and life skills education in schools; and (3) responses with very good levels of efficacy, including family and parenting skills training as well as screening and brief interventions.

**Fig. 1:**
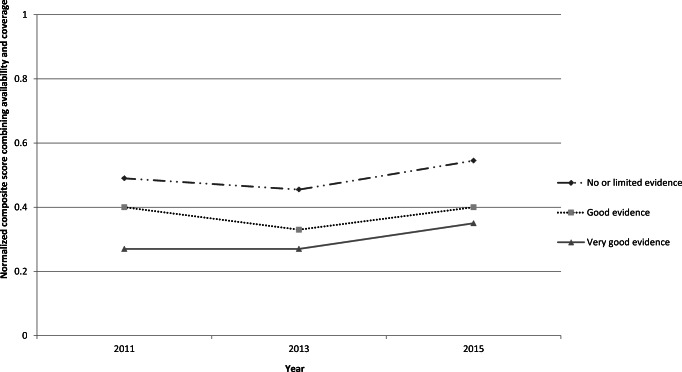
Trends in availability and coverage of prevention (combined to a normalized composite score) by reporting year of the ARQ part II, stratified by level of evidence of effectiveness per the International Standards on Drug Use Prevention

A composite score was then generated to summarize the information about the actual implementation of a given type of prevention (yes/no) and, if yes, at what level of coverage of the target population (low, medium, or high). This score for the level of implementation was generated for each of the three strength-of-evidence categories and was normalized between 0 and 1 to enable cross-comparison (United Nations Economic and Social Council 2015).^2^ This score was intended to measure the degree of implementation of evidence-based prevention globally and to illustrate trends in the coverage of prevention approaches (1) not supported by solid evidence, (2) supported with good evidence, and (3) supported with very good evidence. These trends over the 10 years since the 2009 Political Declaration are presented in Fig. 1.

#### Results of Analyzing the Global State of Prevention Practices

The prevention interventions with no or limited evidence for their effectiveness were reported to have higher coverage than the interventions supported by good levels of evidence across all three timepoints. Even when the reported availability and coverage across all categories of prevention approaches increased from 2013 to 2015, the globally predominant prevention responses remained to be those not in line with the evidence (Fig. 1).

While the ARQ offers data on the availability and coverage of different types of global prevention activities, the survey does have limitations. It does not ask whether the member states are implementing their prevention interventions based on tested protocols or are implementing other types of prevention activities not listed in the provided menu of options. In addition, the response rate to the ARQ influences the extent and quality of information in the analysis. While 70% of the member states (60 countries) provided the ARQ part II data in all three biannual cycles reported in this article, these respondents were mostly from Europe, Asia, and the Americas (42%, 30%, and 21% of the responses, respectively), with only a few responses from Africa. Thus, the ARQ data do not allow us to draw solid conclusions about the trends in prevention programming, and the data are not necessarily representative of all geographical regions. Additional work remains to be done to further develop this data collection instrument because this tool remains the only source of information depicting the global state of prevention programming and the translation of prevention research into practice.

Finally, the data gathered with this instrument also reveal a significant gap in evaluation. The ARQ shows that a majority of the reported prevention activities (55%) were not evaluated at all in 2010–2011, and process evaluation was far more common than outcome evaluation for those activities reported to be evaluated. For example, only 1.9% of the member states implementing life skills education in schools reported evaluating its impacts (United Nations Economic and Social Council 2012). These findings illustrate a scarcity in both implementing prevention approaches supported by evidence and implementing them with rigor and a sufficient understanding of their efficacy in their given context. Furthermore, these findings validate the view that the capacities of national decision-makers in prevention planning and evaluation require support.

### Prioritization and Conceptualization of Prevention in the UNODC’s Policy Documents Guiding Global Action to Address Drug Use

#### Materials and Methods for Analyzing the Global Frameworks Guiding Prevention

Resolutions are the formal documents through which the CND formulates a consensus on how governments, as well as the UNODC, should address the global drug use problem. The CND meets and issues such resolutions yearly. The sections of these resolutions calling for member states, the UNODC, and other international organizations to take concrete actions are called the operational paragraphs. They constitute the so-called mandates, or political commitments and internationally shared goals for international and national action on drug-related matters, including prevention. The other sections of these documents provide the contexts and justifications for these mandates.

**Fig. 2:**
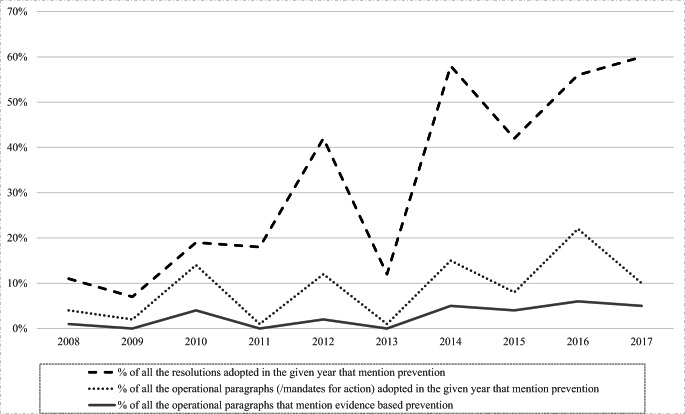
The percentages of the resolutions, and of their operational paragraphs, adopted by the CND between 2008 and 2017, that call for prevention of substance use and for evidence-based prevention approaches

The text of all the resolutions adopted by the CND from 2009 to 2017 (UN 2010–2017) was reviewed to provide a concise overview of how these resolutions portrayed the prioritization of prevention within the continuum of health and law enforcement responses to drug use. This analysis also looked at how the language deployed in these resolutions reflected conceptualizations of what constitutes desirable prevention. One author and a research assistant independently conducted content analysis to identify which paragraphs referred to substance use prevention, and their results had no significant discrepancies. First, to determine what proportion of these mandates and goals for international action addressed substance use (among the wide array of other supply and demand reduction issues), we calculated the percentage of resolutions adopted in a given year that referred to prevention at least once (black dotted line in Fig. 2). Next, we calculated the proportion of the operational paragraphs calling for prevention (gray dotted line in Fig. 2). Finally, we conducted content analysis of how the language used in the operational paragraphs referred to prevention. The number of times that prevention was referred to as evidence-based prevention (sentences referring to the UNODC’s standards, evidence- or science-based prevention, or the evaluation of prevention) was counted by the author and cross-checked by the research assistant. The proportion of operational paragraphs that specifically called for evidence-based prevention was calculated (the gray solid line in Fig. 2). Although rudimentary, this typology was created to illustrate the conceptualizations of desirable prevention held by the highest-level policymakers negotiating these consensus documents.

#### Results of Analyzing the Global Frameworks Guiding Prevention

The proportion of annual resolutions addressing substance use prevention has increased over the years. While prevention was not among the top priorities during the first years following the adoption of the 2009 Political Declaration, the resolutions have more strongly reflected the importance of prevention during 2013–2017. During the years 2008–2012, an average of 20% of the resolutions referred to prevention. The mean percentage of resolutions referring to prevention increased to 45% over 2013–2017 (black line in Fig. 2; section 2 in Table 1). In the operational paragraphs—the section of the CND resolutions providing concrete mandates for action—a similar, slightly positive trend can be observed, although an even smaller minority of them focused on prevention (gray dotted line in Fig. 2). Finally, when looking more closely at how the mandates described prevention, our analysis shows that only a small part of the mandates was dedicated to evidence-based prevention, despite a slightly upward trend from a mean of 1% from the total of the operational paragraphs of the resolutions adopted during 2008–2012 to a mean of 4% of adopted operational paragraphs during 2013–2017 (gray solid line in Fig. 2; section 4 of Table 1). The analyses reveal that, in the political frameworks guiding actions on the global drug problem, evidence-based prevention has not been optimally represented and has been left in the shadow of other health- and law enforcement-related issues.

**Table 1:** The counts and percentages of the resolutions and of their operational paragraphs, adopted by the CND between 2008 and 2017, that call for prevention of substance use in general, and that call particularly for evidence-based approaches

1. Resolutions and their operational paragraphs per year			2. Resolutions that mention prevention			3. Operational paragraphs that mention prevention			4. Operational paragraphs that mention evidence-based prevention			
Year	*N* of all the resolutions adopted in the given year	*N* of all the operational paragraphs of the resolutions adopted in the given year	*N* of the resolutions that mention prevention of substance use adopted in the given year	% out of all the resolutions adopted in that year	Mean %	*N* of the operational paragraphs mentioning the prevention of substance use adopted in the given year	% out of all the operational paragraphs of the resolutions adopted in that year	Mean %	*N* of the operational paragraphs that mention evidence-based prevention*	% out of all the operational paragraphs of the resolutions adopted in that year	Mean %	
2017	10	170	6	60%	45%	17	10%	11%	9	5%	4%	
2016	9	185	5	56%		40	22%		11	6%		
2015	12	144	5	42%		12	8%		6	4%		
2014	12	113	7	58%		17	15%		6	5%		
2013	17	171	2	12%		1	1%		0	0%		
2012	12	108	5	42%	19%	13	12%	7%	2	2%	1%	
2011	17	136	3	18%		1	1%		0	0%		
2010	16	120	3	19%		17	14%		5	4%		
2009	14	118	1	7%		2	2%		0	0%		
2008	18	113	2	11%		5	4%		1	1%		

^*^Occasions when prevention is referred to as “evidence-based” or “science-based,” or when evaluation of effectiveness or scientific assessment is called for, or when the implementation of the UNODC Standards is called for

## The UNODC’s Global Program on Prevention: Top-down and Bottom-up Approaches

The UNODC’s current global prevention program (summarized in the supplemental resource) is two-pronged. Global or top-down components target policymakers, while a national or bottom-up approach focuses on piloting evidence-based prevention interventions in specific countries to demonstrate their feasibility and benefits and to gradually create capacities for scaling up. The outcomes of the UNODC’s grassroots-level work have been reported elsewhere (Maalouf and Campello 2014; Mejia et al. 2015; 2016). The top-down approach centers on facilitating readiness and capacity among national decision-makers. These efforts include informal bilateral communication, advocacy, publication of guidance documents intended to tailor and package evidence for the needs of policymakers, and training activities aimed at fostering dialog and building their capacities to support evidence-based prevention. The International Standards on Drug Use Prevention (UN 2015), which summarize the available scientific evidence on what constitutes an effective prevention response, forms the core guidance document underpinning all of the UNODC’s actions on prevention.

The UNODC’s top-down efforts involve training through which the UNODC engages directly with policymakers in particular countries or regions. The aim is to influence their attitudes and knowledge regarding the nature and potential value of evidence-based prevention, including its evaluation. Since the publication of the International Standards on Drug Use Prevention (UN 2015), the UNODC has reached more than 1000 policymakers and other national stakeholders. The first wave of this training initiative helped decision-makers think critically about their national prevention responses in light of their needs, their resources, and the current state of evidence as summarized in the standards. The previously reported evaluation of this training initiative demonstrated its ability to influence the knowledge and attitudes of policymakers (Campello et al. 2016).

## The UNODC’s Efforts to Build Policymakers’ Capacities and Create Readiness for Prevention Evaluation

UNODC organized four follow-up regional capacity building seminars to continue the dialog with policymakers with the aims to strengthen national prevention responses and to increase policymakers’ capacities specifically related to evaluation. These seminars reached nearly 90 decision-makers from 30 LMICs. As in the previous training, the participants were nominated by their governments and represented the different governmental sectors in charge of prevention in their countries. These 2-day interactive training seminars were intended to increase these national-level policymakers’ appreciation of the value of evaluating substance use prevention activities. Seminars discussed the benefits of grounding their planning in rigorous data collection and existing research and upholding their roles in supporting a culture of evaluation. The seminars also promoted overall appreciation of the essential role of prevention and its potential to support public health. To nurture dialog on research, policy, and practice across the different sectors in charge of prevention within the targeted countries, the seminars were also aimed at supporting wider shifts in the cultural and normative contexts within which the participants operated. Detailed information on how to conduct evaluations of effectiveness was not provided because it was not the role of these policymakers to conduct evaluations themselves. Instead, the seminars discussed the various ways in which these policymakers could support evaluation research and utilize the outcomes of evaluation studies to advance the prevention field. The seminars also reviewed the different study designs and types of evaluations, the role of evaluation in the project cycle, and the roles of the various stakeholders involved in evaluation in prevention programming. The training showcased existing resources and highlighted positive examples of successful evaluations. Each of these topics was taught via lectures introducing key related concepts and interactive exercises.

## Results of the Policymakers’ Capacity Building Exercise

An assessment was conducted with anonymous pre- and post-test questionnaires to determine changes in the participants’ (1) knowledge of the key learning goals of the training; (2) attitudes concerning the usefulness of evaluation; and (3) self-efficacy to utilize research for their professional benefit. Data were collected from participants in three of the regional workshops. A total of 55 participants from 25 countries returned both the pre- and the post-questionnaire, and the changes between the pre- and post-measurements were analyzed. The number of answers to each individual question varied because many respondents left some blank answers.

### Changes in Knowledge About the Types of Evaluation Research

The questionnaire first asked the participants to read different examples of evaluation study results and to identify the types of evaluation research that might produce such results (feasibility studies, monitoring of activities, cost-benefit analysis, or evaluation of effectiveness). The aim was to assess whether the training had helped the participants comprehend the different applications of evaluations correctly (Table 2). Before the training, the majority (80%) of the attendees were able to correctly identify cost-benefit analyses. However, identifying feasibility studies was challenging (only 11% identified them correctly), and the participants commonly confused them with evaluations of effectiveness. After the training, the participants were more cognizant of the requirements of evaluations used to assess the effectiveness of prevention activities, as well as the evaluation types that merely signaled feasibility. Overall, the mean proportion of correct answers increased from 47.9 to 62.2% from the pre-test to the post-test, indicating statistically significant improvement (*p* < 0.05; 29.8%; Table 2).

**Table 2:** Correct identification of different types of evaluations by policymakers attending “Regional training seminar for policymakers on evaluation of the effectiveness of drug use prevention” between 2015 and 2017 (*N* = 55)

Type of prevention***	Pre-training		Post-training		*P* (_*X*_^2^)	Delta change (*B*−*A*)	Relative delta change ((*B*−*A*)/*A*) × 100
	N1*	*A*—proportion of correct answers (SD)**	N2*	*B*—proportion of correct answers (SD)**			
Feasibility study	4	0.11 (0.05)	14	0.28 (0.06)	0.066	0.17	154%
Cost-benefit analysis	29	0.80 (0.65)	45	0.88 (0.45)	0.475	0.08	10%
Evaluation of effectiveness	23	0.50 (0.07)	35	0.69 (0.06)	0.676	0.19	38%
Monitoring of activities	21	0.51 (0.08)	37	0.76 (0.06)	0.023	0.25	49%
Monitoring of activities/feasibility study	10	0.29 (0.08)	27	0.54 (0.07)	0.026	0.25	86%
Total		0.45 (0.03)		0.63 (0.03)	0.033	0.18	40%

*Number of correct answers

**The proportion of correct answers among those who provided an answer to the given question (range 0–1)

***The questionnaire asked the participants to connect the following sentences on possible evaluation outcomes to the type of evaluation that might produce them: “A vast majority of the facilitators stated the program is easy to use, and reported observing lower levels of problematic behavior among the children after the program” (feasibility study); “The program is estimated to save 35 dollars for every dollar invested in the future health and social care as well as criminal justice costs” (cost-benefit analysis); “After the program, the initiation of marijuana use was significantly lower in the intervention group than in the control group” (evaluation of effectiveness); “Activities reached a total of 582 individuals” (monitoring); “80% of the participants stated they are either “satisfied” or “very satisfied” with the program outcomes” (feasibility study and monitoring were both accepted as correct answers)

### Changes in Attitudes and Self-efficacy

The policymakers were also presented with different statements to assess their perceptions of the potential benefits of evaluating prevention and to measure their self-efficacy to conduct and use evaluations in ways benefitting their professional tasks and roles. The participants were asked to indicate to what extent they agreed with each statement (rated on a Likert scale of 1–5), and the results are presented in Table 3. Self-efficacy in knowing how to plan and manage high-quality evaluations of effectiveness increased over the course of the training (from a mean of 2.52 to 3.50, resulting in a 39.4% relative change in the standardized means). A statistically insignificant improvement was noted in perceptions of how easy and cost-beneficial evaluation could be. Perceptions of the possibilities of achieving monetary savings via evaluation did not change from pre- to post-test (Table 3). These results point to the potential of such training to support readiness for more evidence-based, rigorous prevention programming.

**Table 3:** Mean scores responses on different statements covering different types of evaluations by policymakers attending “Regional training seminar for policymakers on evaluation of the effectiveness of drug use prevention” between 2015 and 2017 (*N* = 55)

Statement	Pre-training		Post-training		*P* value (*t* test)	Delta change (*B*−*A*)	Relative delta change (*B*−*A*)/*A*
	*A*		*B*				
	N1	Mean (SD)	N2	Mean (SD)			
Evaluating prevention activities can be easy and not costly compared with the tangible benefits it brings	47	3.26 (1.19)	49	3.53 (1.40)	0.286	0.27	8.2%
I know how to plan and manage a good quality evaluation assessing the effectiveness of prevention program	47	2.51 (0.93)	49	3.50 (1.03)	0.010	0.99	39.4%
Evaluating effectiveness of prevention is a realistic way to achieve monetary savings	47	3.08 (1.38)	48	3.08 (1.35)	0.996	0	0.0%

_Mean scores on a Likert scale (strongly disagree (1) to strongly agree (5)_

## Discussion

The previous discussions and research point to the need to translate and transfer the knowledge derived from the abundant prevention-related research into the complex environments of real-world policies and programs and to promote readiness for and appreciation of evidence-based prevention globally, particularly in LMICs (Fishbein et al. 2016; Patel et al. 2013; Parra-Cardona et al. 2018; Spoth et al. 2013). The presented data from the UNODC sources confirm the existence of such a global knowledge–practice gap.

To describe the translation of evidence at the level of prevention practices, we used data from the UNODC ARQ, which is currently the only source of information on the status of prevention activities implemented globally. The analysis of the data shows a scarcity of systematic, rigorous prevention implementation. First, prevention activities not supported by evidence are still the type most commonly implemented on a global scale. Second, only a minority of the reported prevention activities are being evaluated, and still fewer are evaluated for their efficacy or effectiveness.

To illustrate the translation of evidence at the level of international guiding policy frameworks, we also conducted content analysis of the UNODC’s policy documents guiding international action on prevention. The results show that prevention, particularly evidence-based prevention, is not optimally prioritized in the international political agenda. However, some positive trends can be observed in the growing attention generated by prevention among other health- and law enforcement-related topics. Although rudimentary, the picture derived from these analyses offers useful heuristics for assessing the global situation of a culture of prevention and demonstrating the need to continue efforts to support the translation of prevention science. The findings call attention to the need to support the translation of evidence at the level of global practices and policy frameworks.

Against this background, we sought to answer what types of actions can be taken to increase decision-makers’ endorsement, adoption, and ongoing support of evidence-based preventive interventions by discussing the UNODC’s work to address substance use globally. We focused on the UNODC’s efforts to build national policymakers’ capacity to support evidence-based substance use prevention. This work represents one example of translation efforts during the last stages of translation—efforts that have received little coverage in translational research. We introduced the results from training intended to develop readiness to evaluate prevention among national policymakers in LMICs. The positive results emerging from the UNODC’s direct engagement with policymakers in the first wave of capacity building for evidence-based prevention (Campello et al. 2016) are complemented by the results of the policymaker training presented in this article. Based on these results, this short, unindividualized, follow-up policymaker training, specifically focused on the value of the evaluation of prevention, could feasibly affect the knowledge and attitudes of the participants. The knowledge and attitudes of policymakers that influence their decision-making on prevention are seen to constitute a key layer on which to build a strong culture of prevention. This positive change in the knowledge and attitudes of the policymakers observed over the course of the training points to the ability of this activity to support the advancement of a global culture of prevention as part of larger efforts required to truly impact the prevention practices at a global scale.

## Limitations

Based on the available data, it is not feasible to accurately analyze changes in the quality and scope of global prevention programming. Further developing data collection on global prevention practices to obtain a more precise picture of them is an important challenge in finding tools to bridge the global knowledge–practice gap. Moreover, it is beyond the scope of this article and the UNODC’s current program of work to analyze the impacts of the UNODC’s training activities on possible changes to national-level prevention practices and, even more so, prevention practices at the global level. However, the instruments used to collect data on changes at the level of trained policymakers could be developed further, for example, to better capture their intentions and to develop a more sophisticated picture of their readiness to support evidence-based prevention.

## Recommendations for Strengthening a Culture of Prevention

### Improved Instrumentation to Assess Prevention Efforts in the UN Context

Better data on the implementation of global prevention responses are needed. To intensify and optimally target translation efforts, it is necessary to have a more precise picture of the knowledge–practice gaps that exist globally. The UNODC ARQ, the only existing instrument used to collect such data on a global scale, offers a valuable but limited view of the quality and extent of prevention implementation globally. Moreover, the data are not geographically representative. The instrument should be further developed to ask for more detailed information to better reflect the contents, quality, and coverage of prevention. Efforts to encourage UN member states to appreciate the value of participating in this data collection exercise and to build their capacity to monitor prevention efforts (such as the capacity building exercises described in this article) should be continued.

### Improved Monitoring of Progress in Prevention in National Contexts

In the Sustainable Development Goal 3.5, UN member states have committed to strengthen the treatment and prevention of substance use. It has created invaluable momentum to advance prevention practices globally. However, the two indicators used to measure progress in achieving this goal by 2030 focus on the availability of treatment and on the prevalence of harmful alcohol use, and do not reflect the availability or quality of prevention practices (UN DESA n.d.). This is a serious impediment to encouraging wider investment and support for prevention. National monitoring systems should overcome this challenge by monitoring the quality and reach of prevention activities implemented nationally and tracking any potential advancements made. National stakeholders should be encouraged and supported to further develop their monitoring systems in the field of substance use prevention. Finally, national authorities should be urged to use and communicate these results to encourage other countries to invest in high-quality prevention efforts.

### Increased Adoption and Sustainability of Evidence-Based Preventive Interventions

Work to advocate for rigorous goals and commitments and to promote prevention efforts higher on the global policymaking agenda should be continued and broadened. During the 2019 CND, representatives of the UNODC member states set new goals to address substance use globally. This effort provides important momentum to strengthen the dialog between the scientific and political communities and to capitalize on the latest scientific knowledge about what works best to address substance use prevention. Capacity building is another key component of advancing evidence-based prevention. Various capacity building activities targeting prevention practitioners, coordinators, and decision-makers have been implemented globally, for example in the context of Universal Prevention Curriculum,^3^ and these complement the efforts to build readiness toward evidence-based prevention among national-level decision-makers. This article presents one example of a capacity building activity for decision-makers and presents the argument that such capacity building can be a tool to enhance policymakers’ readiness to support evidence-based prevention, particularly in LMICs. Such efforts to increase decision-makers’ endorsement, adoption, and ongoing support of evidence-based preventive interventions should be continued and intensified.

## Electronic supplementary material

ESM 1 (DOCX 22 kb)

## Footnotes

1 To collect information on coverage, the questionnaire asks respondents to select whether the given intervention reaches a “low,” “medium,” or “high” “proportion of the target population in need of an intervention” based on their assessment of the situation.

2 The score would be 0 if all the respondents reported not implementing any of the approaches in this category and 1 if all the reporting countries reported implementing all of these approaches with high coverage.

3 Universal Prevention Curriculum for Substance Use at the International Society of Substance Use Prevention and Treatment Professionals website: https://www.issup.net/training/universal-prevention-curriculum; and the European Prevention Curriculum at the European Monitoring Centre for Drugs and Drug Addiction website: http://www.emcdda.europa.eu/best-practice/european-prevention-curriculum_en.
