# Supplementary material for: The United Nations Office on Drugs and Crime’s Efforts to Strengthen a Culture of Prevention in Low- and Middle-Income Countries
Source: Prev Sci. 2020 Jan 17;22(1):18–28. doi: 10.1007/s11121-020-01088-5 (PMC7762745; doi:10.1007/s11121-020-01088-5)
Supplement: Supplementary file 1 — (DOCX 22 kb) [file 11121_2020_1088_MOESM1_ESM.docx]

Supplemental table. UNODC global framework of operation for advancing evidence-based prevention programming

| Tool | Purpose | Mode of activities | Objective in advancing the culture of prevention |
| --- | --- | --- | --- |
| Top down model of engagement with policy makers to advocate for a better-informed decision-making and evidence-based prevention | | | |
| Annual Report Questionnaires (ARQ) Part II | Monitor the implementation of ‘the Political Declaration and Plan of Action on International Cooperation towards an Integrated and Balanced Strategy to Counter the World Drug Problem’ adopted by UN Member States in 2009 | Analysis of responses bi-annually  Reporting to the Commission on Narcotic Drugs on type of prevention interventions implemented globally, their level of coverage and the degree evaluation. | Showcase the status of prevention programming globally and advocate for attenuation of non-evidence prevention response while increasing focus on the evidence-based ones |
| The International Standards on Drug Use Prevention | Define the etiological construct upon which a drug prevention strategy should be based on.  List and describe the types interventions and policies that have been found to be efficacious in preventing substance abuse, together with the characteristics that appear to be linked to positive, negative or no prevention outcomes. | Published guidance (2013), dissemination and advocacy efforts | Guide the UNODC work on prevention including in the headquarters (global framework of operations) as well as in the field offices (country and regional programmes and projects not described here);  Guide the Member States in their prevention programming (including via the political mandates referring to the Standards) |
| Other guidance documents | Provide guidance on specific topics and for specific target groups | Publish and disseminate guidance documents, i.e. on family skills programming (2009); for education sector (2017), for those working in rural settings (2017) | Encourage and build capacities for wider evidence-based programming |
| Capacity building among national decision makers | Increase the understanding of the national level policymakers of the value and importance of evidence-based prevention programmes and policies in addressing substance use related problems | ‘Policy Makers and Prevention’ – training seminars based on International Prevention Standards (2013-2015)  Training seminars on the value and importance of evaluating the effectiveness of substance use prevention (2016-2017) | Encourage more evidence-based programming creating capacities and readiness for it; encourage increasing the volume of research available from low- and middle-income countries; encourage data collection on substance use and prevention, including in the context of ARQ |
| Advocacy efforts | Encourage evidence-based prevention programming creating readiness for it | Advocacy campaigns: ‘Listen First’ (2016) and the annual world drug day campaign  Annual advocacy and discussion events on evidence-based prevention for delegates in the contexts of the UN political meetings | Encourage more evidence-based programming |
| Bilateral support for the UNODC Member States | Support and guide the Member States in the national prevention planning and in the related international political processes | Bilateral meetings and consultations  Formal consultation/ technical support in the UN policy making context (in negotiations on resolutions concerning substance use and its prevention) | Directly support the strengthening of the national prevention responses  Support in creating political commitments and mandates in the UN context |
| ‘Bottom-up’ approach | | | |
| Piloting evidence-based prevention programs under GLOK01 -project | Encourage policy makers to adopt evidence-based prevention programmes as part of their national strategic response and provide the with means for scaling up and sustaining them | Adapt and pilot evidence-based prevention programmes (focusing on family skills and on life skills in school contexts) in low- and middle-income countries in co-operation with the Member States | Showcase that evidence based approached are transferable, feasible to implement and effective in addressing substance use related problems |
